# Eastern Equine Encephalitis Virus Exhibits More Charged Amino Acids in Its Envelope Proteins, Compared to Madariaga Virus

**DOI:** 10.4269/ajtmh.24-0693

**Published:** 2025-08-07

**Authors:** Gladys Medina, Jose Luis Zambrano, Carmen Luisa Loureiro, Domingo J. Garzaro, Miguel Barrios, Rosa Alba Salas, Scott C. Weaver, Flor H. Pujol

**Affiliations:** ^1^ Edificio de Sanidad Animal, Laboratorio de Arbovirus-CENIAP/INIA, Aragua, Venezuela;; ^2^ Laboratorio de Virología Molecular, CMBC, Instituto Venezolano de Investigaciones Científicas (IVIC), Caracas, Venezuela;; ^3^ Laboratorio de Virología Celular, CMBC, Instituto Venezolano de Investigaciones Científicas (IVIC), Caracas, Venezuela;; ^4^ Residencias Thamara, Caracas, Venezuela;; ^5^ World Reference Center for Emerging Viruses and Arboviruses, Institute for Human Infections and Immunity and Department of Microbiology and Immunology, University of Texas Medical Branch, Galveston, Texas

## Abstract

Given the importance of eastern equine encephalitis virus (EEEV; complex lineage I) in veterinary and public health in North America, there is limited knowledge about lineages II, III, and IV, also known as Madariaga virus (MADV), which are prevalent in Central and South America. Because charge mutations in the envelope glycoproteins are critical to the emergence of Venezuelan equine encephalitis virus, we analyzed the structural polyproteins of Venezuelan MADV isolates and compared them with sequences of EEEV. Four substitutions involving positively charged residues were identified in the E3 (R44Q) and E2 (R205H/Q, K260T/A, and R310Q) proteins. Additionally, histidine residues were present in EEEV and absent in MADV: E2 (H82Y, H94Y, H114Q, and H181S) and E1 (H118Q). None of these amino acids were predicted to be under selective pressure or associated with a significant conformational change in the envelope proteins. However, some of these substitutions might still be associated with the virulence and pathogenicity of EEEV.

## INTRODUCTION

Eastern equine encephalitis virus (EEEV; species *Alphavirus eastern*; lineage I in the eastern equine encephalitis [EEE] complex) produces a wide spectrum of clinical signs and symptoms in equids and humans, ranging from undifferentiated febrile illness to central nervous system (CNS) involvement and death, with both human and equine case fatality rates exceeding 30%. The EEE complex also includes Madariaga virus (MADV; species *Alphavirus madariaga*, EEE complex lineages II, III, and IV), which was formerly considered a South American variant of EEEV.^1^ Lineage III is the most widely distributed throughout the Americas, from Panama to Argentina. Epizootic outbreaks of MADV occur periodically, causing high mortality in equine populations and sometimes CNS disease in humans, as described in Brazil, Haiti, Trinidad and Tobago, and Venezuela, as well as in a significant outbreak in Panama. However, they generally result in a lower mortality rate among humans.^1^ Like EEEV outbreaks, these MADV outbreaks are self-limiting because of the lack of high viremia in terminal hosts (equids and humans).

Studies involving other alphaviruses, such as Venezuelan equine encephalitis (*Alphavirus venezuelan*), have revealed that differences in the charge of the surface glycoprotein E2 are associated with phenotypic variation, including virulence. Additionally, mutations resulting in positively charged residues can lead to emergence.^2^ Similarly, it has been demonstrated that many alphaviruses, including Sindbis virus (SINV), Ross River virus, and Venezuelan Equine Encephalitis Virus (VEEV), adapt to bind heparan sulfate (HS) through mutations encoding positively charged amino acids after successive passages in cell cultures.^3^ To determine if charged amino acids differ in MADV compared with EEEV, possibly explaining differences in virulence, translated subgenomic RNA sequences were compared across lineages I, II, III, and IV of the EEE complex to identify virulence determinants potentially associated with positively charged amino acids.

The sequences of the structural polyprotein open reading frames of 11 MADV isolates from Venezuela were determined, as previously described,^4^ and compared with sequences from the four lineages of the EEE complex available in GenBank (Supplemental Table 1). A total of 448 complete genome sequences of EEEV and complete genomes (*n* = 38) or structural polyprotein coding regions (*n* = 9) of MADV were available in GenBank as of January 6, 2025. The sequences of EEEV (*n* = 448) and MADV (*n* = 58: 47 in GenBank and 11 from this study) were aligned using MAFFT (https://mafft.cbrc.jp/alignment/server/index.html) and translated with MEGA11 (MEGA Software, Dortmund, Germany).^5^^,^^6^ Madariaga virus amino acid sequences were then analyzed for charged amino acids and histidines in MADV, compared with EEEV.

**Figure 1.**
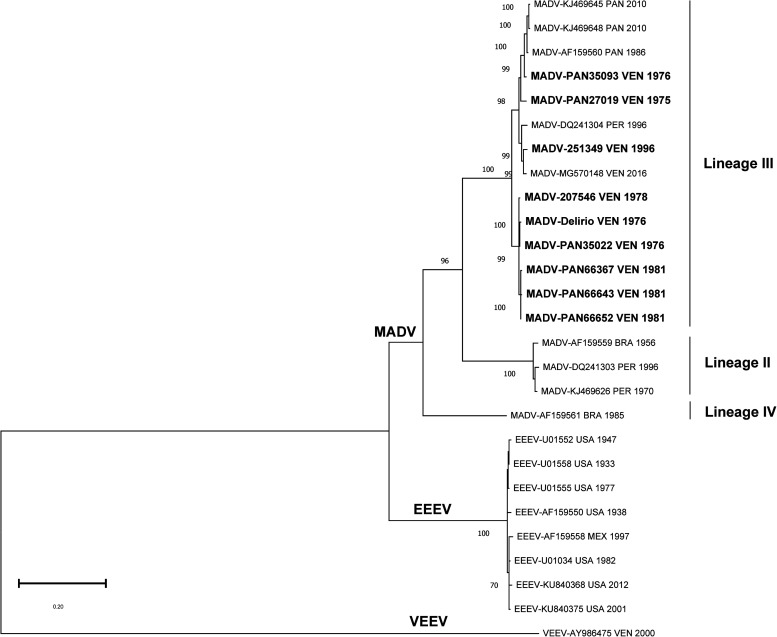
Phylogenetic tree for the eastern equine encephalitis virus complex. The region encoding for the structural proteins was analyzed. Evolutionary history was inferred by using the maximum likelihood method and the general time-reversible model.^5^ The tree with the highest log likelihood (–19,846.22) is shown. The percentage of trees in which the associated taxa clustered together is shown next to the branches. Initial trees for the heuristic search were obtained by applying the neighbor-joining method to a matrix of pairwise distances estimated using the maximum composite likelihood approach. A discrete Gamma distribution was used to model evolutionary rate differences among sites (four categories: +G; parameter = 0.33320). The tree is drawn to scale, with branch lengths measured in the number of substitutions per site. This analysis involved 27 nucleotide sequences. The codon positions included first, second, third, and noncoding regions. All positions with less than 90% site coverage were eliminated (i.e., fewer than 10% alignment gaps, missing data), and ambiguous bases were allowed at any position (partial deletion option). A total of 3,735 positions were included in the final dataset. Evolutionary analyses were conducted using MEGA11 (MEGA Software, Dortmund, Germany).^5^ The sequences in bold represent the Venezuelan Madariaga virus sequenced in this study.

A phylogenetic tree of the sequenced MADV strains is shown in Figure 1. All Venezuelan isolates belonged to MADV lineage III. Using a sequence of VEEV as an outgroup, EEEV exhibited an ancestral position relative to the three MADV lineages.

Our comparison of 506 sequences from the four EEE complex lineages, including an analysis of 1,242 amino acids, revealed the following MADV differences relative to EEEV:

- Loss of charged residues in E3 (R44Q) and E2 (R205H/Q, K260T/A and R310Q);
- Loss of histidine residues, which can behave as proton donors, in E2 (H82Y, H94Y, H114Q, and H181S) and E1 (H118Q/L).

Some minor exceptions were observed: in total, 11/506 sequences contained unique substitutions, with 5/448 EEEV and 6/57 MADV exhibiting these changes (Supplemental Table 1). Of the six MADV sequences with unique amino acids, five belonged to lineage II (K205) and one belonged to lineage IV (R310).

The role of charged amino acids in EEEV pathogenesis remains unknown. The fact that the enzootic transmission cycles differ between VEE and EEE complexes (primarily rodents for VEEV and probably MADV; birds for EEEV) suggests that viral emergences may follow different evolutionary trajectories.

The presence of positive charges in *Alphavirus* envelope proteins may indicate artifactual selection for HS binding during cell culture passages.^3^ We discounted this possible artifact because our analyses included 24 EEEV isolates directly sequenced from human cases (comprising complete or nearly complete genome sequences, from 1983 to 2023; GenBank KJ469587, KJ469603, KP282670, KU059747, KX000164, OQ709265-OQ709280, and OR988088). Almost all the charged residues described above were conserved among these strains. No increase in positively charged amino acids was observed in the sequences of MADV infecting humans in Panama and Haiti (GenBank OR644805, MH359230, MH359231, MH359232, and MH359233).

The differences we found in the number of histidine residues could affect virulence and the differential pathogenicity of these alphaviruses. Lee et al. found that single mutations in glycoprotein E2 of SINV were important for neurovirulence and receptor binding. Specifically, histidine 55 in E2 played an important role due to its binding to neuronal cells.^7^

Recent studies have revealed that the very low-density lipoprotein receptor (VLDLR) acts as a receptor for both EEEV and MADV.^8^ Almost all the specific amino acids involved in the interaction with this receptor are conserved in the EEE complex. For the EF151503 sequence (lineage IV, Br85), two motifs in E2 associated with interaction with VLDLR exhibited substitutions compared with all other EEE complex sequences: L2V and T4A in K232R and W234R. However, these substitutions were generally conservative.

To determine if any of the amino acids studied were subjected to selective pressure, an analysis was performed with Datamonkey,^9^ using three different methods: Fixed effects likelihood, mixed effects model of evolution, and single-likelihood ancestor counting. Only two amino acids were identified by all three methods: A45 of the EEEV E6 polypeptide and T209 of the MADV E2 glycoprotein. None of the charged amino acids nor the histidines appeared to be under positive selection.

**Figure 2.**
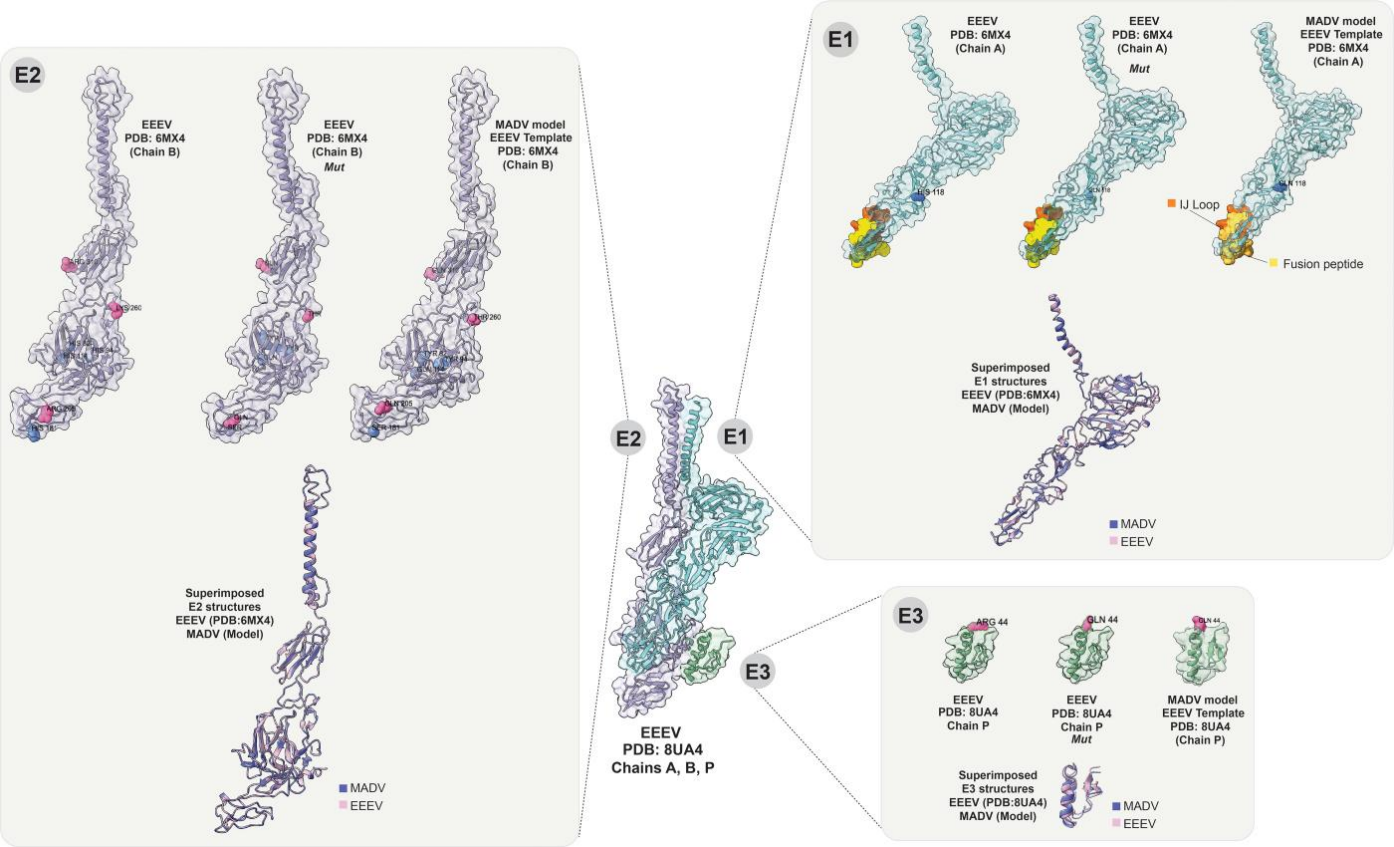
Homology modeling of Madariaga virus (MADV) and mutated eastern equine encephalitis virus (EEEV) envelope protein structures. Ribbon diagram of E1 (cyan, chain A), E2 (purple, chain B), and E3 (green, chain P) from the crystal structure of EEEV viral-like protein (Protein Data Bank: 8UA4) depicting the crystal conformation of the three EEEV proteins. Madariaga virus E1 and E2 protein structures were modeled using the SWISS-MODEL servers (Expasy, Geneva, Switzerland), based on the 6MX4 PDB (chain A and chain B, respectively). 6MX4 is a cryo-electron microscopy structure of chimeric EEEV used as a template. Madariaga virus E3 protein was modeled using the SWISS-MODEL servers, based on the 8UA4 PDB (chain P). The ij loop (orange) and fusion peptide (yellow) are shown in the E1 protein structures. Colored residues represent Arg residue losses in MADV (pink), and His residue losses in MADV (blue). The 6MX4 and 8UA4 PDB and homology structures were visualized and analyzed, and amino acid residues were mutated with the rotamers tool by using UCSF ChimeraX (version 1.9; University of California San Francisco, San Francisco, CA). Sequence identities included E1: 90.23% (global model quality estimation: 0.73; qualitative model energy analysis: 0.67 ± 0.5), E2: 87.35% (QMQE: 0.74; QMEAN 0.65 ± 0.5), and E3: 96.30 (QMQE: 0.68; QMEAN 0.70 ± 0.11; 9–11). In addition, mutated structures of the EEEV envelope proteins were modeled, where the positively charged residues and histidines described in this study (shown in blue in E1, pink in E2, and red in E3) were replaced with the corresponding amino acids present in the MADV envelope proteins (EEEV Mut). Beneath each structure is the superimposed structure of EEEV and MADV proteins.

Structural modeling of the envelope proteins^10^^–^^12^ revealed no significant differences between EEEV and MADV, except for minor changes observed in the E3 protein. Replacing ancestral histidines and charged residues of EEEV with MADV amino acids did not alter the structure of any of the in silico mutated envelope proteins of EEEV. The superimposed figure of the structure of each viral protein shows an almost perfect alignment (Figure 2).

In conclusion, a significant reduction in charged amino acids was observed in MADV envelope proteins compared with ancestral EEEV sequences. Positively charged amino acids have been associated with significant changes in pathogenicity (such as in epizootic VEEV strains and other attenuated alphaviruses). Whether these charges are related to the increased pathogenicity observed in EEEV compared with MADV requires further study.

## Supplemental Materials

10.4269/ajtmh.24-0693 Supplemental Materials
